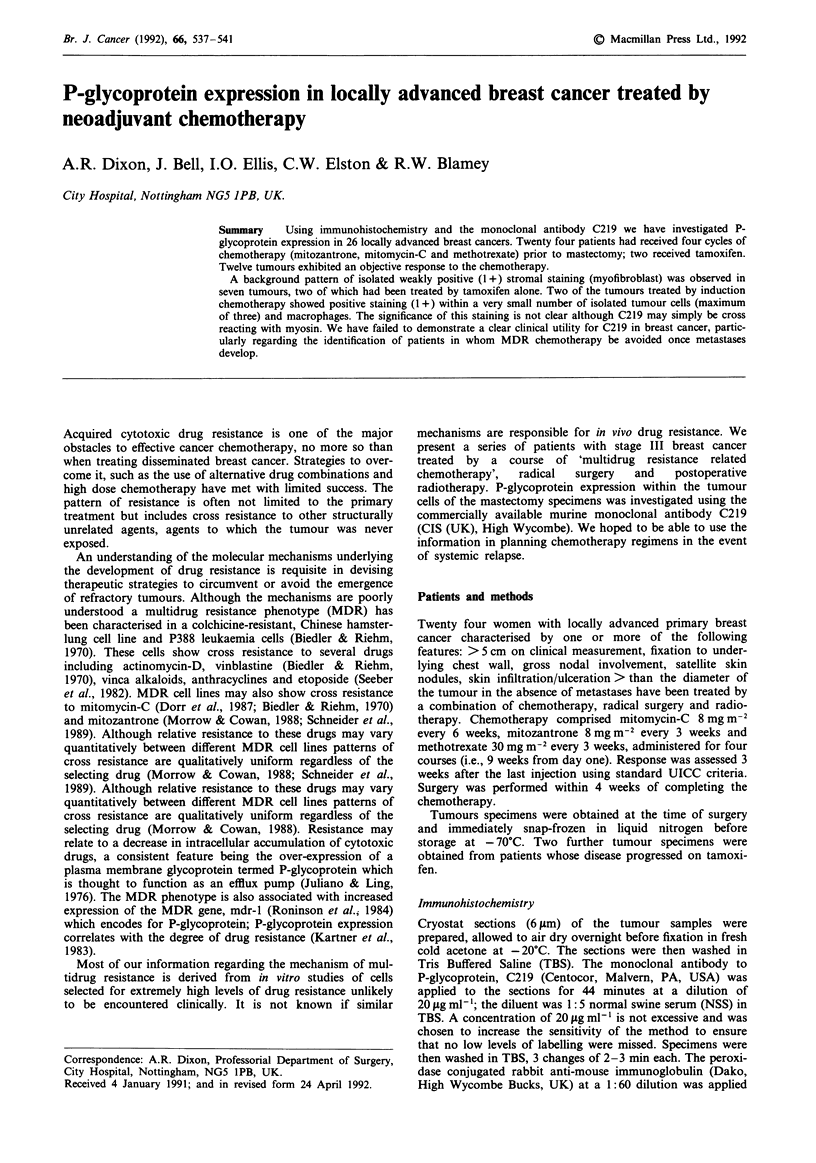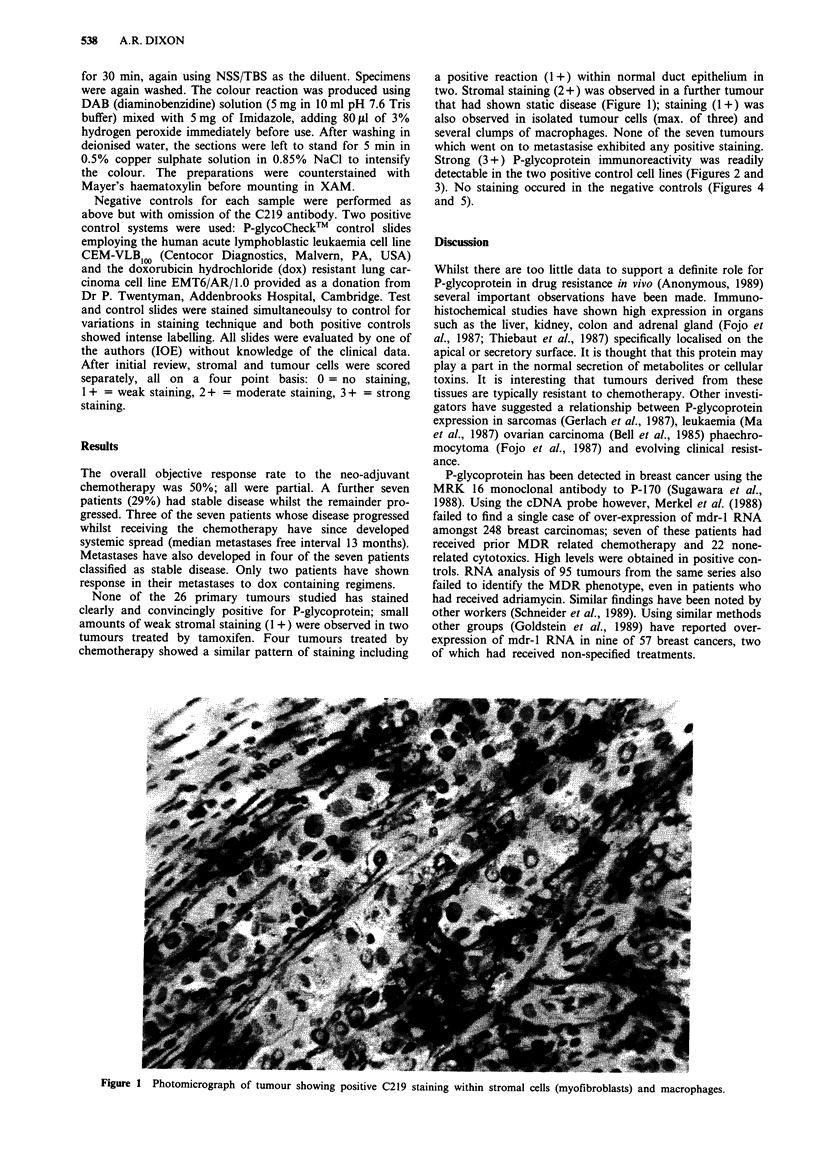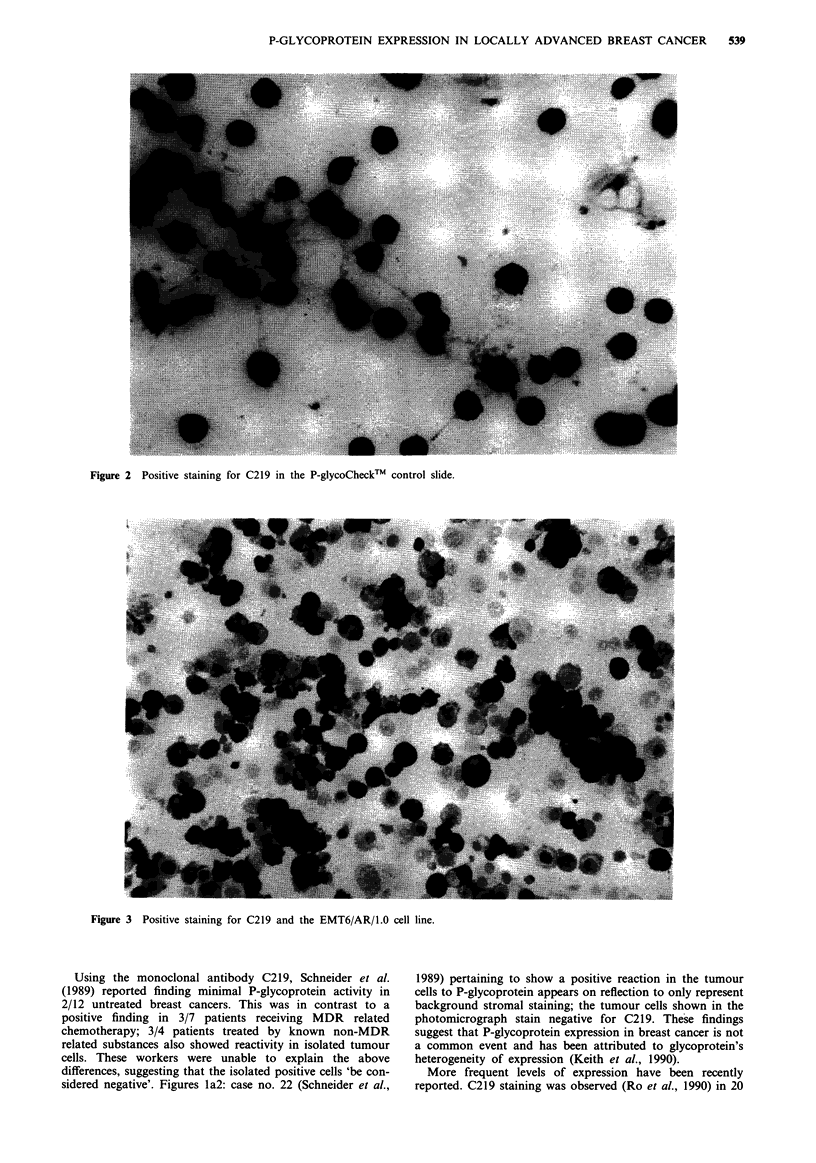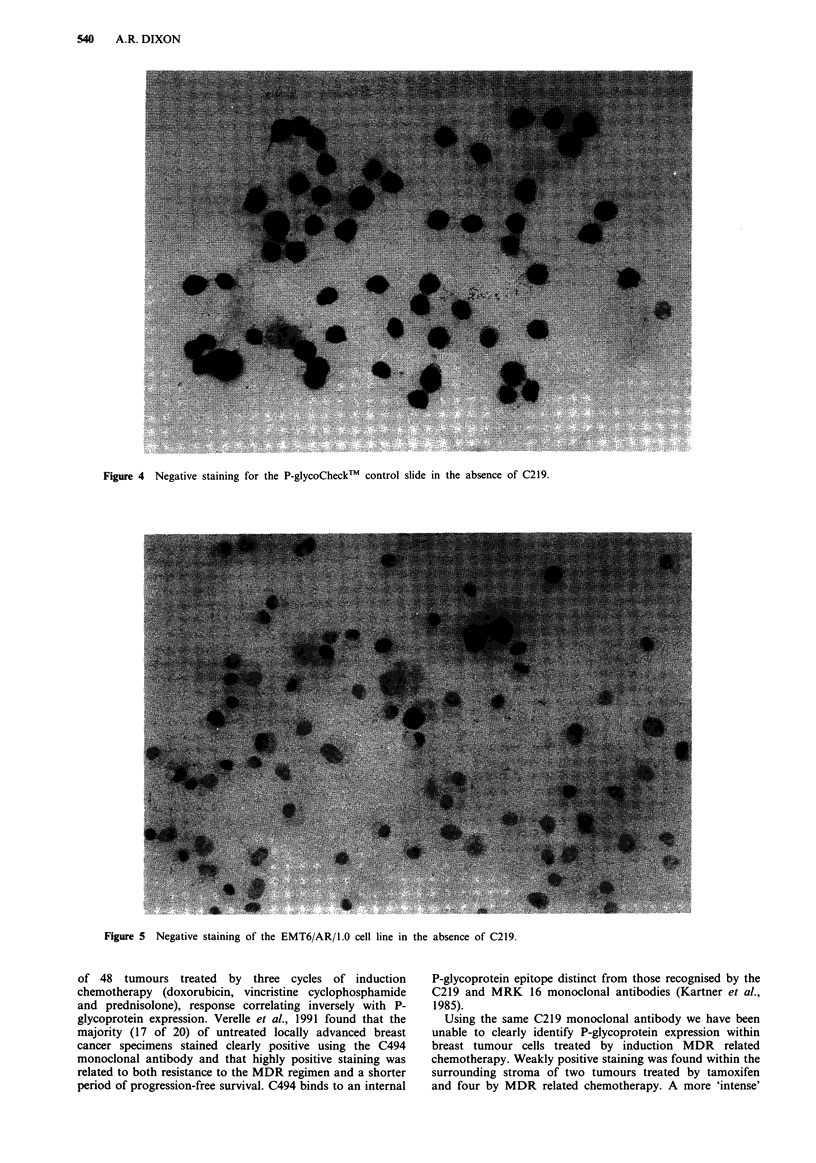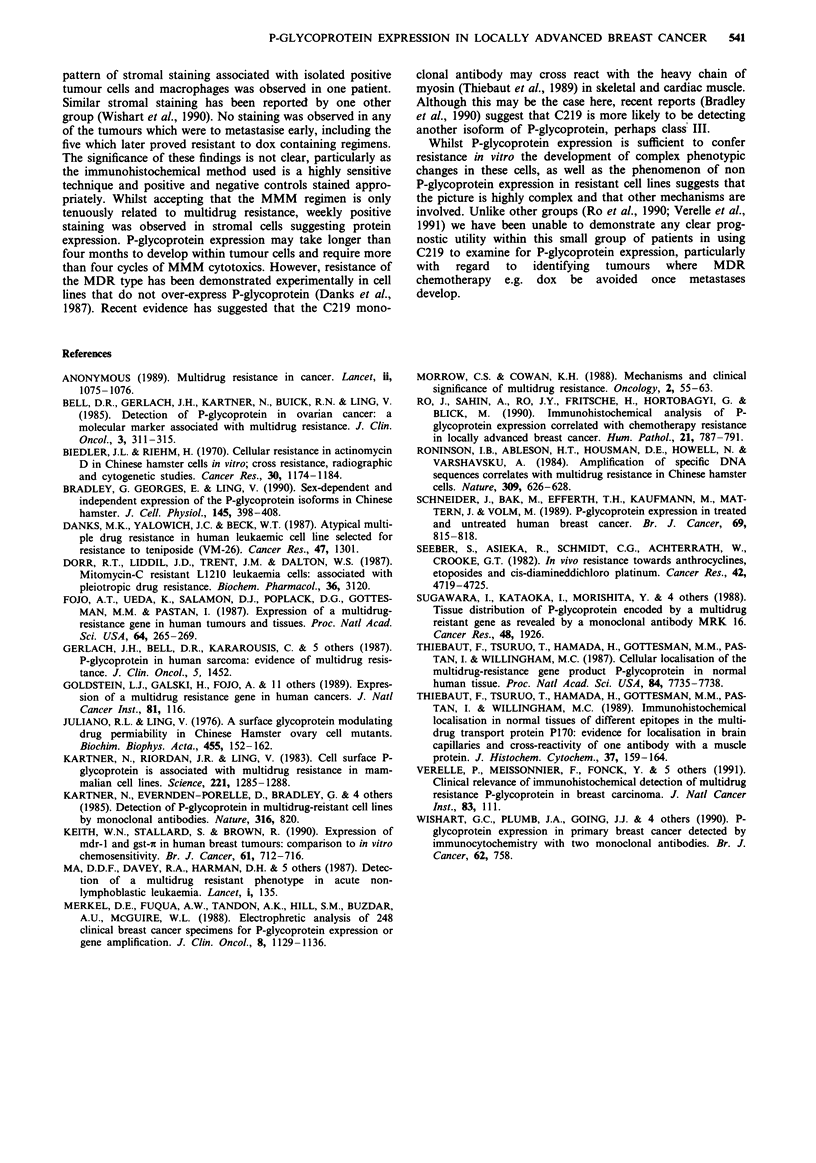# P-glycoprotein expression in locally advanced breast cancer treated by neoadjuvant chemotherapy.

**DOI:** 10.1038/bjc.1992.309

**Published:** 1992-09

**Authors:** A. R. Dixon, J. Bell, I. O. Ellis, C. W. Elston, R. W. Blamey

**Affiliations:** City Hospital, Nottingham, UK.

## Abstract

**Images:**


Br. J. Cancer (1992), 66, 537–541 © Macmillan Press Ltd., 1992

### P-glycoprotein expression in locally advanced breast cancer treated by neoadjuvant chemotherapy

A.R. Dixon, J. Bell, I.O. Ellis, C.W. Elston & R.W. Blamey

*City Hospital, Nottingham NG5 1PB, UK.*

**Summary** Using immunohistochemistry and the monoclonal antibody C219 we have investigated P-glycoprotein expression in 26 locally advanced breast cancers. Twenty four patients had received four cycles of chemotherapy (mitozantrone, mitomycin-C and methotrexate) prior to mastectomy; two received tamoxifen. Twelve tumours exhibited an objective response to the chemotherapy.

A background pattern of isolated weakly positive (1+) stromal staining (myofibroblast) was observed in seven tumours, two of which had been treated by tamoxifen alone. Two of the tumours treated by induction chemotherapy showed positive staining (1+) within a very small number of isolated tumour cells (maximum of three) and macrophages. The significance of this staining is not clear although C219 may simply be cross reacting with myosin. We have failed to demonstrate a clear clinical utility for C219 in breast cancer, particularly regarding the identification of patients in whom MDR chemotherapy be avoided once metastases develop.

Acquired cytotoxic drug resistance is one of the major obstacles to effective cancer chemotherapy, no more so than when treating disseminated breast cancer. Strategies to overcome it, such as the use of alternative drug combinations and high dose chemotherapy have met with limited success. The pattern of resistance is often not limited to the primary treatment but includes cross resistance to other structurally unrelated agents, agents to which the tumour was never exposed.

An understanding of the molecular mechanisms underlying the development of drug resistance is requisite in devising therapeutic strategies to circumvent or avoid the emergence of refractory tumours. Although the mechanisms are poorly understood a multidrug resistance phenotype (MDR) has been characterised in a colchicine-resistant, Chinese hamster-lung cell line and P388 leukaemia cells (Biedler & Riehm, 1970). These cells show cross resistance to several drugs including actinomycin-D, vinblastine (Biedler & Riehm, 1970), vinca alkaloids, anthracyclines and etoposide (Seeber *et al.*, 1982). MDR cell lines may also show cross resistance to mitomycin-C (Dorr *et al.*, 1987; Biedler & Riehm, 1970) and mitozantrone (Morrow & Cowan, 1988; Schneider *et al.*, 1989). Although relative resistance to these drugs may vary quantitatively between different MDR cell lines patterns of cross resistance are qualitatively uniform regardless of the selecting drug (Morrow & Cowan, 1988; Schneider *et al.*, 1989). Although relative resistance to these drugs may vary quantitatively between different MDR cell lines patterns of cross resistance are qualitatively uniform regardless of the selecting drug (Morrow & Cowan, 1988). Resistance may relate to a decrease in intracellular accumulation of cytotoxic drugs, a consistent feature being the over-expression of a plasma membrane glycoprotein termed P-glycoprotein which is thought to function as an efflux pump (Juliano & Ling, 1976). The MDR phenotype is also associated with increased expression of the MDR gene, mdr-1 (Roninson *et al.*; 1984) which encodes for P-glycoprotein; P-glycoprotein expression correlates with the degree of drug resistance (Kartner *et al.*, 1983).

Most of our information regarding the mechanism of multidrug resistance is derived from *in vitro* studies of cells selected for extremely high levels of drug resistance unlikely to be encountered clinically. It is not known if similar mechanisms are responsible for *in vivo* drug resistance. We present a series of patients with stage III breast cancer treated by a course of 'multidrug resistance related chemotherapy', radical surgery and postoperative radiotherapy. P-glycoprotein expression within the tumour cells of the mastectomy specimens was investigated using the commercially available murine monoclonal antibody C219 (CIS (UK), High Wycombe). We hoped to be able to use the information in planning chemotherapy regimens in the event of systemic relapse.

### Patients and methods

Twenty four women with locally advanced primary breast cancer characterised by one or more of the following features: >5 cm on clinical measurement, fixation to underlying chest wall, gross nodal involvement, satellite skin nodules, skin infiltration/ulceration > than the diameter of the tumour in the absence of metastases have been treated by a combination of chemotherapy, radical surgery and radiotherapy. Chemotherapy comprised mitomycin-C 8 mg m$^{-2}$ every 6 weeks, mitozantrone 8 mg m$^{-2}$ every 3 weeks and methotrexate 30 mg m$^{-2}$ every 3 weeks, administered for four courses (i.e., 9 weeks from day one). Response was assessed 3 weeks after the last injection using standard UICC criteria. Surgery was performed within 4 weeks of completing the chemotherapy.

Tumours specimens were obtained at the time of surgery and immediately snap-frozen in liquid nitrogen before storage at −70°C. Two further tumour specimens were obtained from patients whose disease progressed on tamoxifen.

### *Immunohistochemistry*

Cryostat sections (6 µm) of the tumour samples were prepared, allowed to air dry overnight before fixation in fresh cold acetone at −20°C. The sections were then washed in Tris Buffered Saline (TBS). The monoclonal antibody to P-glycoprotein, C219 (Centocor, Malvern, PA, USA) was applied to the sections for 44 minutes at a dilution of 20 µg ml$^{-1}$; the diluent was 1:5 normal swine serum (NSS) in TBS. A concentration of 20 µg ml$^{-1}$ is not excessive and was chosen to increase the sensitivity of the method to ensure that no low levels of labelling were missed. Specimens were then washed in TBS, 3 changes of 2–3 min each. The peroxidase conjugated rabbit anti-mouse immunoglobulin (Dako, High Wycombe Bucks, UK) at a 1:60 dilution was applied

Correspondence: A.R. Dixon, Professorial Department of Surgery, City Hospital, Nottingham, NG5 1PB, UK.
Received 4 January 1991; and in revised form 24 April 1992.

for 30 min, again using NSS/TBS as the diluent. Specimens were again washed. The colour reaction was produced using DAB (diaminobenzidine) solution (5 mg in 10 ml pH 7.6 Tris buffer) mixed with 5 mg of Imidazole, adding 80 µl of 3% hydrogen peroxide immediately before use. After washing in deionised water, the sections were left to stand for 5 min in 0.5% copper sulphate solution in 0.85% NaCl to intensify the colour. The preparations were counterstained with Mayer's haematoxylin before mounting in XAM.

Negative controls for each sample were performed as above but with omission of the C219 antibody. Two positive control systems were used: P-glycoCheck™ control slides employing the human acute lymphoblastic leukaemia cell line CEM-$VLB_{100}$ (Centocor Diagnostics, Malvern, PA, USA) and the doxorubicin hydrochloride (dox) resistant lung carcinoma cell line EMT6/AR/1.0 provided as a donation from Dr P. Twentyman, Addenbrooks Hospital, Cambridge. Test and control slides were stained simultaneoulsy to control for variations in staining technique and both positive controls showed intense labelling. All slides were evaluated by one of the authors (IOE) without knowledge of the clinical data. After initial review, stromal and tumour cells were scored separately, all on a four point basis: 0 = no staining, 1+ = weak staining, 2+ = moderate staining, 3+ = strong staining.

### Results

The overall objective response rate to the neo-adjuvant chemotherapy was 50%; all were partial. A further seven patients (29%) had stable disease whilst the remainder progressed. Three of the seven patients whose disease progressed whilst receiving the chemotherapy have since developed systemic spread (median metastases free interval 13 months). Metastases have also developed in four of the seven patients classified as stable disease. Only two patients have shown response in their metastases to dox containing regimens.

None of the 26 primary tumours studied has stained clearly and convincingly positive for P-glycoprotein; small amounts of weak stromal staining (1+) were observed in two tumours treated by tamoxifen. Four tumours treated by chemotherapy showed a similar pattern of staining including a positive reaction (1+) within normal duct epithelium in two. Stromal staining (2+) was observed in a further tumour that had shown static disease (Figure 1); staining (1+) was also observed in isolated tumour cells (max. of three) and several clumps of macrophages. None of the seven tumours which went on to metastasise exhibited any positive staining. Strong (3+) P-glycoprotein immunoreactivity was readily detectable in the two positive control cell lines (Figures 2 and 3). No staining occured in the negative controls (Figures 4 and 5).

### Discussion

Whilst there are too little data to support a definite role for P-glycoprotein in drug resistance *in vivo* (Anonymous, 1989) several important observations have been made. Immunohistochemical studies have shown high expression in organs such as the liver, kidney, colon and adrenal gland (Fojo *et al.*, 1987; Thiebaut *et al.*, 1987) specifically localised on the apical or secretory surface. It is thought that this protein may play a part in the normal secretion of metabolites or cellular toxins. It is interesting that tumours derived from these tissues are typically resistant to chemotherapy. Other investigators have suggested a relationship between P-glycoprotein expression in sarcomas (Gerlach *et al.*, 1987), leukaemia (Ma *et al.*, 1987) ovarian carcinoma (Bell *et al.*, 1985) phaechromocytoma (Fojo *et al.*, 1987) and evolving clinical resistance.

P-glycoprotein has been detected in breast cancer using the MRK 16 monoclonal antibody to P-170 (Sugawara *et al.*, 1988). Using the cDNA probe however, Merkel *et al.* (1988) failed to find a single case of over-expression of mdr-1 RNA amongst 248 breast carcinomas; seven of these patients had received prior MDR related chemotherapy and 22 none-related cytotoxics. High levels were obtained in positive controls. RNA analysis of 95 tumours from the same series also failed to identify the MDR phenotype, even in patients who had received adriamycin. Similar findings have been noted by other workers (Schneider *et al.*, 1989). Using similar methods other groups (Goldstein *et al.*, 1989) have reported over-expression of mdr-1 RNA in nine of 57 breast cancers, two of which had received non-specified treatments.

**Figure 1** Photomicrograph of tumour showing positive C219 staining within stromal cells (myofibroblasts) and macrophages.

**Figure 2** Positive staining for C219 in the P-glycoCheck™ control slide.

**Figure 3** Positive staining for C219 and the EMT6/AR/1.0 cell line.

Using the monoclonal antibody C219, Schneider *et al.* (1989) reported finding minimal P-glycoprotein activity in 2/12 untreated breast cancers. This was in contrast to a positive finding in 3/7 patients receiving MDR related chemotherapy; 3/4 patients treated by known non-MDR related substances also showed reactivity in isolated tumour cells. These workers were unable to explain the above differences, suggesting that the isolated positive cells 'be considered negative'. Figures 1a2: case no. 22 (Schneider *et al.*, 1989) pertaining to show a positive reaction in the tumour cells to P-glycoprotein appears on reflection to only represent background stromal staining; the tumour cells shown in the photomicrograph stain negative for C219. These findings suggest that P-glycoprotein expression in breast cancer is not a common event and has been attributed to glycoprotein's heterogeneity of expression (Keith *et al.*, 1990).

More frequent levels of expression have been recently reported. C219 staining was observed (Ro *et al.*, 1990) in 20

**Figure 4** Negative staining for the P-glycoCheck™ control slide in the absence of C219.

**Figure 5** Negative staining of the EMT6/AR/1.0 cell line in the absence of C219.

of 48 tumours treated by three cycles of induction chemotherapy (doxorubicin, vincristine cyclophosphamide and prednisolone), response correlating inversely with P-glycoprotein expression. Verelle *et al.*, 1991 found that the majority (17 of 20) of untreated locally advanced breast cancer specimens stained clearly positive using the C494 monoclonal antibody and that highly positive staining was related to both resistance to the MDR regimen and a shorter period of progression-free survival. C494 binds to an internal P-glycoprotein epitope distinct from those recognised by the C219 and MRK 16 monoclonal antibodies (Kartner *et al.*, 1985).

Using the same C219 monoclonal antibody we have been unable to clearly identify P-glycoprotein expression within breast tumour cells treated by induction MDR related chemotherapy. Weakly positive staining was found within the surrounding stroma of two tumours treated by tamoxifen and four by MDR related chemotherapy. A more 'intense'

pattern of stromal staining associated with isolated positive tumour cells and macrophages was observed in one patient. Similar stromal staining has been reported by one other group (Wishart *et al.*, 1990). No staining was observed in any of the tumours which were to metastasise early, including the five which later proved resistant to dox containing regimens. The significance of these findings is not clear, particularly as the immunohistochemical method used is a highly sensitive technique and positive and negative controls stained appropriately. Whilst accepting that the MMM regimen is only tenuously related to multidrug resistance, weekly positive staining was observed in stromal cells suggesting protein expression. P-glycoprotein expression may take longer than four months to develop within tumour cells and require more than four cycles of MMM cytotoxics. However, resistance of the MDR type has been demonstrated experimentally in cell lines that do not over-express P-glycoprotein (Danks *et al.*, 1987). Recent evidence has suggested that the C219 monoclonal antibody may cross react with the heavy chain of myosin (Thiebaut *et al.*, 1989) in skeletal and cardiac muscle. Although this may be the case here, recent reports (Bradley *et al.*, 1990) suggest that C219 is more likely to be detecting another isoform of P-glycoprotein, perhaps class III.

Whilst P-glycoprotein expression is sufficient to confer resistance *in vitro* the development of complex phenotypic changes in these cells, as well as the phenomenon of non P-glycoprotein expression in resistant cell lines suggests that the picture is highly complex and that other mechanisms are involved. Unlike other groups (Ro *et al.*, 1990; Verelle *et al.*, 1991) we have been unable to demonstrate any clear prognostic utility within this small group of patients in using C219 to examine for P-glycoprotein expression, particularly with regard to identifying tumours where MDR chemotherapy e.g. dox be avoided once metastases develop.

### References

ANONYMOUS (1989). Multidrug resistance in cancer. *Lancet*, **ii**, 1075-1076.

BELL, D.R., GERLACH, J.H., KARTNER, N., BUICK, R.N. & LING, V. (1985). Detection of P-glycoprotein in ovarian cancer: a molecular marker associated with multidrug resistance. *J. Clin. Oncol.*, **3**, 311-315.

BIEDLER, J.L. & RIEHM, H. (1970). Cellular resistance in actinomycin D in Chinese hamster cells *in vitro*; cross resistance, radiographic and cytogenetic studies. *Cancer Res.*, **30**, 1174-1184.

BRADLEY, G. GEORGES, E. & LING, V. (1990). Sex-dependent and independent expression of the P-glycoprotein isoforms in Chinese hamster. *J. Cell. Physiol.*, **145**, 398-408.

DANKS, M.K., YALOWICH, J.C. & BECK, W.T. (1987). Atypical multiple drug resistance in human leukaemic cell line selected for resistance to teniposide (VM-26). *Cancer Res.*, **47**, 1301.

DORR, R.T., LIDDIL, J.D., TRENT, J.M. & DALTON, W.S. (1987). Mitomycin-C resistant L1210 leukaemia cells: associated with pleiotropic drug resistance. *Biochem. Pharmacol.*, **36**, 3120.

FOJO, A.T., UEDA, K., SALAMON, D.J., POPLACK, D.G., GOTTESMAN, M.M. & PASTAN, I. (1987). Expression of a multidrug-resistance gene in human tumours and tissues. *Proc. Natl Acad. Sci. USA*, **64**, 265-269.

GERLACH, J.H., BELL, D.R., KARAROUSIS, C. & 5 others (1987). P-glycoprotein in human sarcoma: evidence of multidrug resistance. *J. Clin. Oncol.*, *5*, 1452.

GOLDSTEIN, L.J., GALSKI, H., FOJO, A. & 11 others (1989). Expression of a multidrug resistance gene in human cancers. *J. Natl Cancer Inst.*, **81**, 116.

JULIANO, R.L. & LING, V. (1976). A surface glycoprotein modulating drug permiability in Chinese Hamster ovary cell mutants. *Biochim. Biophys. Acta.*, **455**, 152-162.

KARTNER, N., RIORDAN, J.R. & LING, V. (1983). Cell surface P-glycoprotein is associated with multidrug resistance in mammalian cell lines. *Science*, **221**, 1285-1288.

KARTNER, N., EVERNDEN-PORELLE, D., BRADLEY, G. & 4 others (1985). Detection of P-glycoprotein in multidrug-reistant cell lines by monoclonal antibodies. *Nature*, **316**, 820.

KEITH, W.N., STALLARD, S. & BROWN, R. (1990). Expression of mdr-1 and gst-π in human breast tumours: comparison to *in vitro* chemosensitivity. *Br. J. Cancer*, **61**, 712-716.

MA, D.D.F., DAVEY, R.A., HARMAN, D.H. & 5 others (1987). Detection of a multidrug resistant phenotype in acute non-lymphoblastic leukaemia. *Lancet*, **i**, 135.

MERKEL, D.E., FUQUA, A.W., TANDON, A.K., HILL, S.M., BUZDAR, A.U., MCGUIRE, W.L. (1988). Electrophretic analysis of 248 clinical breast cancer specimens for P-glycoprotein expression or gene amplification. *J. Clin. Oncol.*, **8**, 1129-1136.

MORROW, C.S. & COWAN, K.H. (1988). Mechanisms and clinical significance of multidrug resistance. *Oncology*, **2**, 55-63.

RO, J., SAHIN, A., RO, J.Y., FRITSCHE, H., HORTOBAGYI, G. & BLICK, M. (1990). Immunohistochemical analysis of P-glycoprotein expression correlated with chemotherapy resistance in locally advanced breast cancer. *Hum. Pathol.*, **21**, 787-791.

RONINSON, I.B., ABLESON, H.T., HOUSMAN, D.E., HOWELL, N. & VARSHAVSKU, A. (1984). Amplification of specific DNA sequences correlates with multidrug resistance in Chinese hamster cells. *Nature*, **309**, 626-628.

SCHNEIDER, J., BAK, M., EFFERTH, T.H., KAUFMANN, M., MATTERN, J. & VOLM, M. (1989). P-glycoprotein expression in treated and untreated human breast cancer. *Br. J. Cancer*, **69**, 815-818.

SEEBER, S., ASIEKA, R., SCHMIDT, C.G., ACHTERRATH, W., CROOKE, G.T. (1982). *In vivo* resistance towards anthrocyclines, etoposides and cis-diamineddichloro platinum. *Cancer Res.*, **42**, 4719-4725.

SUGAWARA, I., KATAOKA, I., MORISHITA, Y. & 4 others (1988). Tissue distribution of P-glycoprotein encoded by a multidrug reistant gene as revealed by a monoclonal antibody MRK 16. *Cancer Res.*, **48**, 1926.

THIEBAUT, F., TSURUO, T., HAMADA, H., GOTTESMAN, M.M., PASTAN, I. & WILLINGHAM, M.C. (1987). Cellular localisation of the multidrug-resistance gene product P-glycoprotein in normal human tissue. *Proc. Natl Acad. Sci. USA*, **84**, 7735-7738.

THIEBAUT, F., TSURUO, T., HAMADA, H., GOTTESMAN, M.M., PASTAN, I. & WILLINGHAM, M.C. (1989). Immunohistochemical localisation in normal tissues of different epitopes in the multidrug transport protein P170: evidence for localisation in brain capillaries and cross-reactivity of one antibody with a muscle protein. *J. Histochem. Cytochem.*, **37**, 159-164.

VERELLE, P., MEISSONNIER, F., FONCK, Y. & 5 others (1991). Clinical relevance of immunohistochemical detection of multidrug resistance P-glycoprotein in breast carcinoma. *J. Natl Cancer Inst.*, **83**, 111.

WISHART, G.C., PLUMB, J.A., GOING, J.J. & 4 others (1990). P-glycoprotein expression in primary breast cancer detected by immunocytochemistry with two monoclonal antibodies. *Br. J. Cancer*, **62**, 758.